# Flux sampling in genome-scale metabolic modeling of microbial communities

**DOI:** 10.1186/s12859-024-05655-3

**Published:** 2024-01-29

**Authors:** Patrick E. Gelbach, Handan Cetin, Stacey D. Finley

**Affiliations:** 1 Alfred E. Mann Department of Biomedical Engineering, University of Southern California, Los Angeles, CA 90089 USA; 2 Department of Quantitative and Computational Biology, University of Southern California, Los Angeles, CA 90089 USA; 3 Mork Family Department of Chemical Engineering and Materials Science, University of Southern California, Los Angeles, CA 90089 USA

**Keywords:** Genome-scale metabolic modeling, Microbial communities, Flux sampling, Cell metabolism

## Abstract

**Background:**

Microbial communities play a crucial role in ecosystem function through metabolic interactions. Genome-scale modeling is a promising method to understand these interactions and identify strategies to optimize the community. Flux balance analysis (FBA) is most often used to predict the flux through all reactions in a genome-scale model; however, the fluxes predicted by FBA depend on a user-defined cellular objective. Flux sampling is an alternative to FBA, as it provides the range of fluxes possible within a microbial community. Furthermore, flux sampling can capture additional heterogeneity across a population, especially when cells exhibit sub-maximal growth rates.

**Results:**

In this study, we simulate the metabolism of microbial communities and compare the metabolic characteristics found with FBA and flux sampling. With sampling, we find significant differences in the predicted metabolism, including an increase in cooperative interactions and pathway-specific changes in predicted flux.

**Conclusions:**

Our results suggest the importance of sampling-based approaches to evaluate metabolic interactions. Furthermore, we emphasize the utility of flux sampling in quantitatively studying interactions between cells and organisms.

**Supplementary Information:**

The online version contains supplementary material available at 10.1186/s12859-024-05655-3.

## Background

Microbes are essential components of all living ecosystems, and the metabolic interactions between them significantly contribute to the function of these ecosystems. Microbe-microbe metabolic interactions affect nutrient cycling, energy production, and the maintenance of microbial diversity [1–3]. Though our understanding of those microbial communities is aided by metagenomics and in vitro analyses, we lack a quantitative, mechanistic understanding of the interactions between members of microbial consortia [4, 5].

Genome-scale modeling has emerged as a promising method by which we can probe an organism's metabolic states, behaviors, and capabilities, either alone or within a community [6–12]. Genome-scale metabolic modeling is a mathematical approach that uses the known biochemical reactions of a species to reconstruct a genome-scale metabolic network. Genome-scale models (GEMs) provide a holistic view of an organism's metabolism and can be applied to gain insight into the metabolic physiological processes. A GEM consists of a stoichiometric matrix that characterizes the interconversion of metabolites by the set of metabolic reactions, linked with a set of Boolean expressions describing the gene-protein-reaction relationships [13].

As a mathematical representation of an organism’s metabolic network, GEMs can be analyzed to predict the flux through all reactions in the network. Furthermore, given the ubiquity of microbial activity in biology, there is substantial value in using metabolic modeling to understand the emergent behaviors and abilities of microbial communities. For example, GEMs have been applied to understand the metabolic interactions of a microbial community in various contexts, including the human gut microbiota and in environmental bioremediation [14–19].

**Fig. 1:**
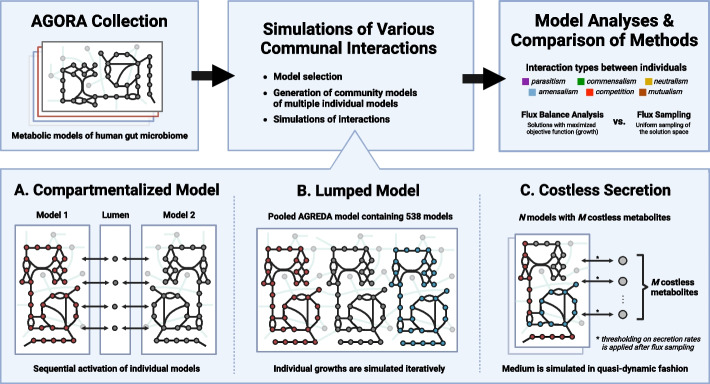
Approaches for genome-scale metabolic modeling of communities. Metabolic modeling of microbial communities is largely performed by selecting metabolic models of the human gut microbiome from AGORA, generating community models using **A** Compartmentalized model: a single stoichiometric matrix representing the two models joined by a lumen compartment wherein metabolites can be freely exchanged; **B** Lumped model: a single stoichiometric matrix representing the union of each individual model’s reactions, thereby ignoring all separation between cells; and **C** Costless secretion: individual stoichiometric networks for each model, whose exchange reactions are constrained to reflect the shared extracellular media

Most metabolic modeling of microbial interactions is performed in one of three ways [20, 21] (Fig. 1): (1) *compartmentalized model*, wherein two metabolic models are merged into a single stoichiometric matrix with a shared compartment representing the extracellular space [22], (2) *lumped model* (also called “enzyme soup”) approach [23–25], where all metabolites and reactions are pooled into a single model in proportion to the community makeup, and (3) *costless secretion*, where models are separately simulated while dynamically and iteratively updating the simulated environment by adjusting the models' exchange reactions and available nutrients based on metabolites that can be secreted without decreasing growth ("costless" metabolites) [26, 27].

Each of these approaches has shown promise, and selection of which approach to use heavily depends on what data and models are available, and on the intended goal of the analysis. As currently implemented, each method uses traditional flux balance analysis (FBA), a linear programming technique that predicts the flow of material through the metabolic network [28–30]. FBA depends on the maximization of an objective function, and maximizing biomass production is most commonly used. Optimizing for biomass assumes species are entirely oriented towards maximal growth, ignoring the multiplicity of achievable sub-optimal phenotypes [31]. When simulating the metabolism of a community, this assumption can disregard the variety of metabolic interactions that the microbes may carry out that do not lead to optimal growth. Furthermore, the selection and definition of the best objective function substantially affect model predictive power and generated results [32–35].

Flux sampling has been used as an alternative to FBA to predict flux distributions. Excitingly, flux sampling may provide a more holistic and accurate description of the cell's flux distribution [36–40]. This is done by randomly generating many flux values for each reaction in a genome-scale metabolic model, while still adhering to defined constraints, such as mass or energy balance and thermodynamic restrictions. Flux sampling employs Markov chain Monte Carlo methods to estimate cellular flux and generate many feasible metabolic flux distributions. Flux sampling estimates the most probable network flux values, enabling statistical comparisons of the flux distributions. Notably, the approach does not require the user to specify a cellular objective, thus reducing user-introduced bias on model predictions and exploring the entire constrained solution space. Flux sampling therefore enables studies of phenotypic heterogeneity, as a single constrained model can generate a range of flux predictions. However, this approach has not been widely employed in analyses of microbial communities. Furthermore, comparisons between FBA-based and sampling-based analyses of communities are currently lacking.

In this work, we apply flux sampling to existing analyses of microbial metabolic interactions, showing the range of potential consortia-wide flux distributions achievable with genome-scale modeling. We find significant differences in model predictions between FBA and flux sampling, with substantial heterogeneity across sampled simulations. We see emergent patterns at sub-maximal growth rates, such as increased cooperation between microbes in anaerobic conditions compared to oxygen-rich environments. In total, we systematically evaluate the impact of flux sampling and emphasize the utility of this approach to study metabolic interactions.

## Methods

### GEMs

Magnúsdóttir et al*.* generated the AGORA dataset, a collection of 773 (and later, 7206 in AGORA2) genome-scale metabolic models comprising the human gut microbiome. These models were simulated to understand their metabolic behavior when grown in pairwise combinations, using the approach developed by Kiltgord and Segre [17, 41, 42]. Notably, the analysis constrained the models with distinct in silico diets and aerobic states, in the presence or absence of oxygen.

We selected 75 of the AGORA models, analyzed all unique pairwise combinations (2775 in total), and implemented three distinct approaches to study metabolic interactions between microbes. In this way, we demonstrate the utility of each approach, compared to flux sampling, while balancing the required computational resources.

### Flux sampling

We use the constrained Riemannian Hamiltonian Monte Carlo (RHMC), which has recently been shown to be substantially more efficient than prior sampling algorithms [43]. Our implementation of the algorithm was accomplished with the COBRA toolbox v3.0 using the Gurobi solver. We used 200 steps per point, 1000 samples per run, parallelized with four workers. The code was simulated in MATLAB, on a 2018 iMac running the MacOS 12 operating system. All uptake rates and media components were left consistent with the original manuscripts, and the sampling approach was maintained with all three techniques.

When generating FBA optimization solutions with which to compare the sampling, we again kept the simulated media (maximum uptake rates) described in the original papers. We note that those prior publications only analyzed a single optimal FBA solution. Therefore, to be more complete, we expanded their baseline analyses by generating an ensemble of equally optimal growth rates with the COBRA Toolbox’s “enumerateOptimalSolutions” function.

### Compartmentalized Modeling

The pairwise interaction approach used by Magnúsdóttir and coworkers is as follows [17]:

- *Step 1*. Select two models.
- *Step 2*. Introduce the lumen compartment, which joins the two models into a merged model such that the two microbes can secrete and uptake metabolites.
- *Step 3*. Constrain the model by adjusting exchange reaction bounds to reflect the chosen diet and extracellular conditions.
- *Step 4*. Simulate monoculture by “shutting off” one of the two models by inactivating all its reactions (setting the reaction flux upper and lower bounds to 0). Then simulate the active individual model by optimizing for growth.
- *Step 5*. Shut off the second individual model by inactivating all its reactions. Then simulate the active individual model by optimizing for growth.
- *Step 6*. Restore the activity of both individuals in the merged model and optimize each model’s growth separately. This predicts growth while allowing the exchange of metabolites across the lumen, simulating co-culture.
- *Step 7*. Compare paired growth with the individual growth simulations of steps 4 and 5. If paired growth was 10% higher or lower than individual growth, the model was considered to grow faster or slower, respectively, in co-culture than alone.

We replaced the FBA optimization in steps 4, 5, and 6 with flux sampling as an alternative way to predict cellular flux. Additionally, we added a basal growth rate for each model, defined as 10% of the model’s individual growth rate achieved by FBA optimization. This ensured a minimum amount of growth and excludes the possibility that one species has no growth at all. We then used the RHMC algorithm and generated 1000 flux distribution samples at each step. We therefore had a range of reaction fluxes (including growth rates) for both microbes, in mono- and co-culture, with and without oxygen, and with two different simulated diets (Western and High Fiber).

### Lumped model

Blasco et al*.* extended the AGORA set of metabolic models by adding degradation pathways that allow for the simulation of the effect of many human diets on the activity of the gut flora [25]. After adding those metabolic reactions involved in degradation, they merged all individual microbe models into a supra-organism model. By pooling all GEMs, they made a single lumped model comprising all metabolic reactions and metabolites in the population.

This method is often called a "mixed-bag" or "bag-of-genes" approach. It is the simplest form of genome-scale modeling of bacterial communities, as it does not assume any separation between the species’ metabolic pathways and involves the consolidation of ubiquitous metabolic reactions [44]–47]. By doing so, the community can be viewed as a single entity, and thus be analyzed with approaches normally applied to single microbial models. Despite its simplicity, the approach has been effective at predicting the metabolic behavior of consortia while minimizing computation time and reducing model size.

The authors used flux variability analysis (FVA) to identify and correct blocked or low-confidence reactions and identify the microbial metabolic byproducts produced by the microbiota's fermentation of lentils. However, the model was not simulated to predict species growth within the community. We therefore used their lumped model and applied the flux sampling method to predict consortia behavior.

With the mixed-bag approach, it is common to merge all individual model biomass reactions into a supra-organism growth equation. Alternatively, one can maintain each model's biomass reaction within the pooled network, as Blasco et al. did. Doing so allows for the prediction of each microbe's growth, in the context of the larger community. Thus, we kept the individual biomass reactions for each species in the model for our analyses and did not create a new lumped biomass representing total community growth.

We generated flux samples of the lumped model and compared them to the case where each species' biomass reaction is optimized alone and to the case where the population's overall growth is maximized. We calculated the “optimal community growth rate” by finding the maximal growth rate that was possible for all models to achieve simultaneously and setting the lower bound of the biomass reaction in each model to that flux value. Thus, the “optimal community growth rate” can be viewed as the individual biomass reactions’ flux predictions with lower bound shared across all biomass reactions. Unfortunately, current computing power and existing algorithms do not allow for optimizing 531 reactions simultaneously (the number of biomass equations in the Blasco lumped model). Therefore, this alternative is the best feasible method to obtain an approximation for optimal community growth.

### Costless secretion

Pacheco et al. showed that the secretion of "costless" metabolites (species that are freely secreted as the byproduct of a cell's metabolism, without inhibiting fitness) are critical drivers of the metabolic interactions between cells [48]. The approach is a quasi-dynamic method, as it maintains the modeling assumption that the system is at a steady state but successively updates the environment shared by the two simulated cells. The method calculates the growth of each model at each simulation iteration, finds the secreted metabolites, then updates the simulated media until the media is stable.

The costless growth approach is as follows:

- *Setup*. Select a simulated minimal media (i.e., DMEM, M9, SSM, etc.), and define the metabolites that comprise that medium. Select *N* models to be simulated (i.e., two models for pairwise interactions, 3 models to simulate a community of 3 microbes, etc.) Select *M* metabolites to be provided alone or in addition to the minimal media (list the carbon sources to be provided, and choose *m* *to be given at a time). Define whether the model will be simulated in an aerobic or anaerobic environment.*
- *Step 1*. Simulate a minimal media condition by setting the upper bound of all exchange reactions to 0 unless the reaction exchanges metabolites that are contained in the media.
- *Step 2*. Provide carbon source(s) *m* by setting the upper bound(s) of exchange reaction(s) for the corresponding metabolite(s) to be unconstrained. We used the same settings used in the costless secretion paper. Namely, growth-limiting carbon sources were set with a max of 10mmol x gDW^-1^ x h^-1^, in order to maintain bounded and feasible growth rates.
- *Step 3*. Simulate the models by optimizing for growth.
- *Step 4*. Record the resulting flux values for the models' transport reactions; if a metabolite is predicted to be exported into the media, then explicitly add that metabolite to the simulated media (again, by adjusting the models' upper bound for that metabolite's import reaction).
- *Step 5*. Repeat steps 3-4 until no additional metabolites are secreted, arriving at the simulation’s final predicted growth rates.
- Steps 1-5 can be repeated for a different carbon source (or combination of carbon sources) added to the simulated media.

We adjusted step 3, replacing FBA optimization with flux sampling using the RHMC algorithm, simulating pairwise growth with a single metabolite source. Because the costless secretion approach repeats the model simulation steps until media convergence, we introduced a thresholding term to consolidate the results from each simulation round. In particular, we define the set of secreted metabolites (and thus update the extracellular media) based on whether all, most, or any of the sampled flux distributions show that a metabolite is secreted. For example, if metabolite *m* was secreted in 200 of the 1000 generated flux distributions, it would only be added to the extracellular media in the “any” cutoff simulation for the next round. If secreted in 750 of the 1000 distributions, it would be added to the “any” and “most” analyses. For the “all” cutoff, that metabolite would only be added to the media for the following iteration if all 1000 flux distributions showed that metabolite being secreted.

Thresholding is currently required because of the computational time needed for flux sampling. Without thresholding at each simulation, the number of sampled points needed will increase exponentially with each round of expansions. We use each cutoff to demonstrate and assess the introduced variability, allowing us to compare the outputs with each set threshold.

We extended the analysis of Pacheco et al*.* by comparing our sampling approach with an ensemble of FBA solutions, as FBA can provide multiple alternative optimal solutions. With the ensemble of optimal FBA solutions, we performed a similar thresholding for adding a metabolite to the next round of simulation.

### Characterizing microbial interactions

**Fig. 2:**
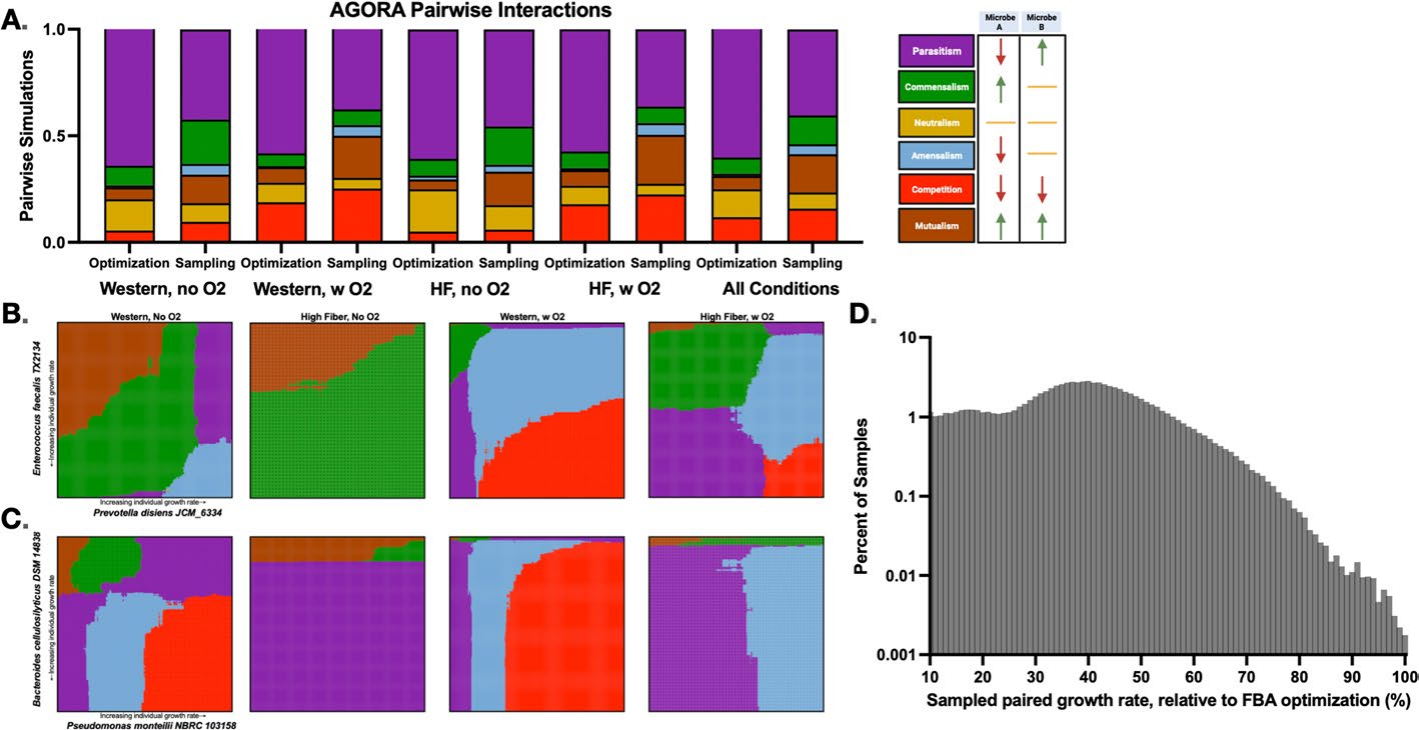
Pairwise analyses of the AGORA/AGORA2 set of models. **A** We simulated 2775 pairs of metabolic models, on two simulated diets, with and without the presence of oxygen, and calculated the expected interaction type. Interactions are defined and colored according to the labels on the far right. Expected interaction type when pairing *Enterococcus* *faecalis* and *Prevotella* *disiens* **B** and *Bacteriodes* *celiilosilyticus* and *Pseudomonas* *montelli* **C** and sampling a range of growth rates. **D** The community growth rate for all pairs considered in (**A**), across all four growth conditions, relative to the pair’s summed growth rate achieved via FBA. Because the requisite basal growth rate for sampling was 10% of the FBA optimal solution, the percent fold change ranged from 10 to 100%

After estimating the reaction fluxes with the three methods described above (with FBA or flux sampling), we categorized all possible combinations of sampled growth rates. Here, we followed the classes previously described: parasitism, commensalism, neutralism, amensalism, competition, or mutualism [17]. The established interaction types compare the individual growth rates of each model with their corresponding growth rates when simulated together. We describe the possible types of interactions below. If both models grew more in co-culture than alone, the interaction was classified as mutualism. If both models grew less in coculture than monoculture, the interaction was classified as competition. If neither model’s growth rate changed when introduced to coculture, the interaction was classified as neutralism. If the first model grows less in co-culture than alone, and the second grew more in co-culture than alone, then the interaction was classified as parasitism. If the first model grows more in co-culture than alone, and the second’s growth was unchanged, then the interaction was classified as commensalism. If the first model grows less in co-culture than alone, and the second’s growth was unchanged, then the interaction was classified as amensalism. The range of possible outcomes are also shown in the figure legend of Fig. 2A.

Additionally, we ordered the sampled growth rates to identify distinct interaction regimes between two microbes. That is, we found the range of different interaction types as a function of the different growth rates. We note that the interaction regimes predicted here are different than the Pareto analysis performed by Magnúsdóttir et al., as calculation of the Pareto front relies on biomass optimization with FBA while iteratively updating and fixing growth rates for each model [17].

## Results

### Compartmentalized modeling

Magnúsdóttir et al. developed AGORA and AGORA2 as resources for semi-automated generation of genome-scale metabolic models [17, 41]. They applied the set of models to predict the pairwise interactions between microbes, showing how the individuals' metabolic potential drives emergent behavior of the pair. However, their predictions of metabolic activity assume that each microbe is oriented towards achieving maximal growth. We introduced flux sampling to the pairwise simulation framework, thus permitting any feasible flux distribution from the confined flux space to be included in the assessment of metabolic interactions. We randomly selected 75 individual models. We then manually augmented the subset of selected models to ensure that the list of 75 models spanned the range of microbial taxa in the AGORA dataset. We considered all possible pairwise combinations of the 75 models, generating 2775 unique pairs. Each set of paired models was sampled 1000 times, with and without metabolite exchange between the microbes being allowed. As described in the Methods, the interaction type was calculated for the anaerobic and aerobic states with two unique simulated diets.

For all combinations of models, we calculated the total percentage of each interaction type, shown in Fig. 2A. As expected, differences exist between the optimization and sampling-based analyses. Namely, antagonistic interactions (competition, amensalism, and parasitism) tend to make up a smaller percentage of the entire set with sampling instead of optimization (61% compared to 74%). There is an increase of 11% in positive and net-neutral interactions (commensalism, neutralism, and mutualism) with sampling compared to optimization-based analysis. Cases of neutralism increased from 6 to 18%, and frequency of mutualism increased from 7 to 13%. The higher frequency of cooperation is particularly prevalent with anaerobic analyses, where cooperative interactions increased from 30 to 44%. Previous work has highlighted that anaerobic conditions induce mutualism; this effect is notably amplified with sampling compared to simulations maximizing biomass [22]. When sampling the possible fluxes of anaerobic conditions, there is a higher frequency of non-inhibitory relationships. In particular, there is a substantial reduction in parasitism with a nearly equivalent increase in neutralism.

Furthermore, we see an increase in symmetrical interactions (mutualism, neutralism, and competition; from 25 to 47%). This result suggests that without orienting all metabolism towards optimal growth, a community of microbial species may be more inclined towards population stability. This is because abundances tend to remain steady when primarily exhibiting those three interaction motifs [49, 50]. Importantly, the trends of increased anaerobic cooperation and increased symmetrical interactions are found irrespective of diet constraints. This suggests that the submaximal predicted growth rate that is allowed with sampling, and not the models themselves or extrinsic factors (such as the nutrients provided), is driving the observed outcomes. In order to ensure that the emergence of the predicted microbial interaction motifs was due to the sampling approach and not to selection of low growth rate pairings or competition that drove one of the species to die off, we calculated the summed growth rates of each model pair considered, and across all four growth conditions. We compared this “community growth rate” for each pair obtained from sampling to that predicted by FBA (Fig. 2D). On average, the summed growth rates predicted with sampling were below that obtained with optimization. However, there were some cases where the results from the sampling approach matched the FBA-derived maximal growth rate.

Because flux sampling gives a distribution of growth rates and the corresponding flux distributions enabling that growth, it is possible to calculate the expected pairwise interactions likely for each combination of individual growth rates. We ranked the sampled growth rates for each microbe and calculated the most commonly predicted paired growth rate, thus giving interaction types for each growth rate. The sampling-based approach highlights the variety of interactions possible between two microbes, especially given variation in simulated conditions. Figure 2 shows this analysis for two sets of paired microbes, represented similar to chemical phase diagrams. The *x*- and *y*-axes represent the individual sampled growth rates, and their intersection is colored according to the most likely expected metabolic interaction motif. Figure 2B shows the pairwise interactions of *Enterococcus faecalis TX2134*, a gram positive nonmotile microbe, and *Prevotella disiens JCM 6632*, a gram negative bacilii-shaped bacterium. Figure 2B shows the interactions of *Bacteriodes celiilosilyticus* and *Pseudomonas montelli*, two gram-negative and rod-shaped microbes. We show these calculations for four distinct extracellular conditions: Western and high fiber diets, with and without oxygen.

When simulating the interactions of the *Enterococcus* and *Prevotella* strains in Fig. 2B, five distinct types of interactions are possible, depending on the simulated environment and each species' growth rate. Anaerobic states (columns 1 and 2) show a predominance of commensalism or mutualism, though parasitism and amensalism are expected when *Prevotella* is rapidly proliferating. In the presence of oxygen (columns 3 and 4), there are several diet-independent trends: low growth of both microbes causes commensalism; high *Enterococcus* and low *Prevotella* growth rates cause parasitism; low *Enterococcus* and high *Prevotella* growth rates cause amensalism; and high growth of both causes competition. At intermediate growth rates, the effect of diet is more apparent, as Western diet constraints drive amensalism and high fiber constraints push the interaction toward commensalism, parasitism, or amensalism.

Similar insights can be gained when analyzing the interactions between *Bacteroides* and *Pseudomonas*. For example, the anaerobic high fiber condition is relatively invariant, as the two microbes show parasitism at nearly all individual growth rates. Alternatively, there is a large set of potential outcomes when simulating an anaerobic state with a Western diet; the individual microbe growth rates can elicit widely distinct interaction motifs. It is possible to see a single microbe "dominate" or drive the observed interaction: in the oxygen-rich simulations, changes in *Pseudomononas* growth determine the outcome, largely independent of the *Bacteriodes* growth rate. Similar analyses can be performed for all combinations of models.

Finally, we propose that this approach can be used to probe the effects of a phenotypically heterogeneous population of genotypically identical microbes. We selected the *Enterococcus faecalis* model and paired it with itself in an anoxic and Western diet-fed environment. This simulates a “clonal population”, and the results are shown in Additional file 1: Fig. S1. The addition of a second copy of the same model generally led to a mutualistic benefit, with the exception of combinations of highly divergent individual growth rates (i.e., near the borders of the plot).

In summary, the sampling-based approach highlights the wide range of predicted interactions possible between two microbes, especially given variety in the simulated conditions.

### Lumped model

By pooling metabolic reactions, it is possible to generate a single GEM that represents community metabolic activity. The lumped GEM can then be analyzed using the same constraint-based approaches typically utilized for single-species models. Though the technique removes all separation between microbes, it can be a useful approach for assessing the activity and potential of the community. However, all analyses of such "bag of genes" or "enzyme soup" approaches have explicitly assumed through the assigning of an objective function, that the community aims to maximize growth. No studies have assessed the effect of flux sampling on the community metabolic state. We selected to study the impact of flux sampling using the AGREDA pooled model, which combined 538 AGORA models into a single metabolic network. We analyzed the lumped model with three distinct approaches: (1) iteratively setting each individual's biomass reaction as the objective and then solving the FBA problem (optimization), (2) performing flux sampling on the network's flux solution space (flux sampling), and (3) finding the maximal rate at which all microbes can simultaneously grow, then sampling the solution space when that value is set as the lower bound for each microbe's growth rate (termed an “optimal community”). These analyses allowed us to compare the flux distributions achieved through FBA, flux sampling, and flux sampling of the “best growth state” of the microbial community.

**Fig. 3:**
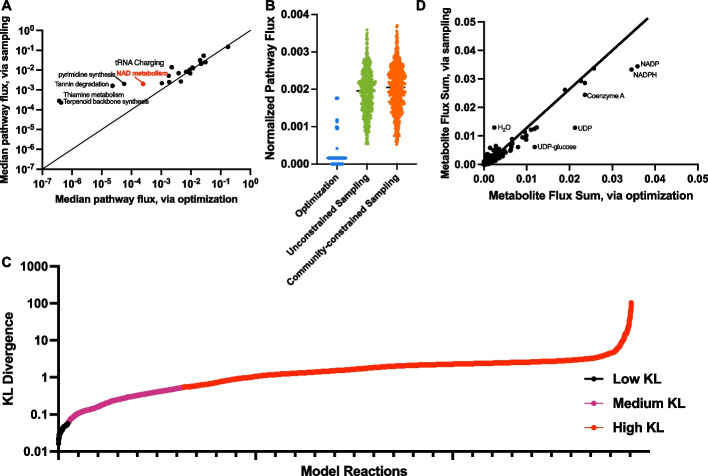
Pooled Model Analyses. **A** Median pathway flux values predicted by unconstrained flux sampling compared to optimization of biomass. Subsystems that have significantly different median fluxes are labeled. **B** Reaction fluxes for the NAD metabolism pathway predicted with each technique. **C** KL divergence between the distribution of fluxes achieved via optimization and sampling. **D** Comparison of the flux-sum value for each metabolite for unconstrained flux sampling and optimization of biomass

When comparing flux sampling of the network with FBA, we first assessed the variation in pathway fluxes to identify large-scale metabolic shifts. Interestingly, flux sampling was not equally influential across all pathways but disproportionately affected particular subsystems. Figure 3A shows the median normalized flux value through each pathway predicted by sampling (*y*-axis) and the median flux value through the pathway when individually optimizing each of the 531 biomass reactions in the model (*x*-axis). That elucidates parts of the network that may be more influential and impactful in community activity when separated from the requirement of maximizing cellular growth. Notably, the thiamine metabolism, terpenoid backbone synthesis, tannin degradation, pyrimidine synthesis, and NAD metabolism pathways had substantially higher fluxes in the sampling approach comp. A similar plot comparing the community-constrained sampling with the FBA approach is shown in Additional file 1: Fig. S2. As an illustrative example, we plot the individual fluxes through the NAD metabolism subsystem obtained by the three techniques used to predict reaction flux (Fig. 3B). Maximization of biomass causes consistently low pathway flux, while unconstrained or optimal community-constrained sampling predicts a wider range of flux values that are higher compared to the fluxes predicted when biomass production is maximized. The case with optimal community-constrained sampling produces slightly elevated pathway flux compared to unconstrained sampling.

At the reaction level, differences emerge between the three community analysis methods. We compared the flux distributions for each reaction, calculating the bi-directional KL divergence values [51] (Fig. 3C). We classified the difference in the median fluxes for each reaction for two analysis methods as low, medium, and high divergence. This calculation revealed that very few (76 of the 5499 reactions; black points) show close alignment between unconstrained flux sampling and optimization of biomass [38, 40]. The predicted flux through a majority of reactions (86%; orange points) is “widely divergent”, between the two approaches. This indicates substantial differences between the optimal growth state and the total solution space. Interestingly, a metabolite-centric view based on median metabolite flux-sum analysis shows similar turnover rates for the metabolites across the two analysis methods (Fig. 3D). We identify a small number of metabolites whose turnover varies widely between the two conditions, including NADP, NADPH, coenzyme A, UDP, and UDP-glucose (higher with optimization) and water (higher with sampling). However, overall, our analyses indicate that while pathway fluxes are higher, the net production or consumption of individual metabolites is approximately the same between FBA- and sampling-based approaches.

### Costless secretion

Pacheco et al. argued that the secretion of "costless" metabolites (byproducts of the cell's metabolism that are secreted without causing a loss of fitness) might be a primary driver of interspecies interactions within a microbial community [48]. In order to study this metabolic cross-feeding, they developed a pipeline where two GEMs are constrained to a minimal media condition, then iteratively simulated, while updating the media with the costless metabolites until convergence. By using FBA, the method assumes that cells grow maximally and that all metabolites secreted enable maximum growth. It is possible that costless metabolites predicted to be secreted depend on the feasible growth rate. Therefore, while the FBA-based is valuable, it may not fully describe the simulated system. By allowing submaximal growth rates and alternative maxima through sampling, we demonstrate increased metabolic latitude for microbial communities.

**Fig. 4:**
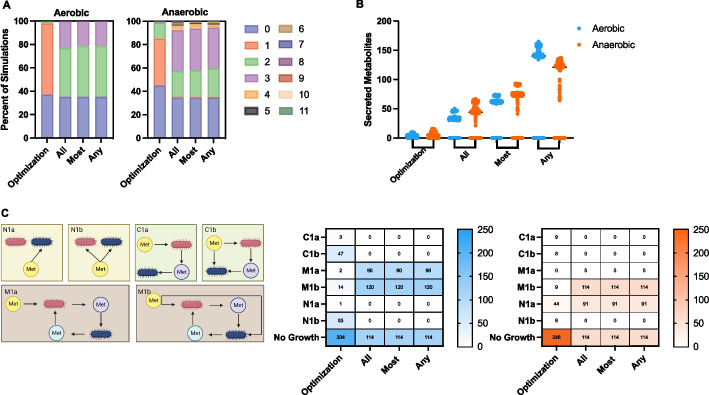
Costless secretion analysis. **A** Number of iterations required to achieve a stable media, for the aerobic (left) and anaerobic (right) states for optimization of biomass or different cutoffs for flux sampling (all, most, any), plotted as a percentage of all simulations. **B** Number of metabolites secreted for optimization of biomass or with distinct cutoffs for flux sampling (all, most, any) for anaerobic and aerobic conditions. **C** Each simulation was categorized into one of 7 cases (the six shown in the left panel and the case where no growth was achieved) for the aerobic or anaerobic condition

A primary output from the costless secretion analysis is the number of iterations of model simulation until media convergence. We simulated 648 cases: pairwise combinations of 3 microbes, with and without oxygen, with 108 distinct fuel sources provided to supplement the minimal media. We assessed the number of iterations required to reach a steady media. Interestingly, for both the aerobic and anaerobic conditions, we see an increase in the number of rounds of model simulation with sampling compared to the base analysis with FBA. This makes sense, as a loosened restriction of growth rate allows for heterogeneous simulation results, which include a greater possible set of metabolites to be secreted and successive changes in the simulated media. That trend of more iterations with sampling remains even when we implement different cutoffs for whether a secreted metabolites is present in all, most, or at least one of the sets of sampled metabolic flux distributions (Fig. 4A). Interestingly, the cutoff selected has much less of an effect than whether FBA (leftmost column) or flux sampling (right three columns) is chosen. We predict a much higher number of iterations in the anaerobic condition, with up to 11 iterations of media change, compared to at most 3 rounds in the aerobic state.

We see an increase in the number of metabolites secreted by the microbe pairs with flux sampling compared to FBA, as shown in Fig. 4B. There is an apparent increase in the number of predicted costless metabolites when the threshold is progressively loosened (from *all* to *most* to *any*). That is reasonable, as there are outliers or secreted metabolites that are particular to one or only few sampled flux distributions. We again predict an increase in secreted metabolites when simulating oxygen-free environments. Specifically, for anerobic conditions, more unique metabolites are secreted as part of the cells' metabolic flux patterns than in the oxygen-rich environments for the two most stringent cutoffs (*all* and *most*).

Pacheco established distinct interaction types, categorized based on the secretion and uptake rates of metabolites, using the following naming convention. The first letter represents the type of interaction: non-interaction (*N*), where no used media metabolites come from either model; commensalism (*C*), characterized by unidirectional exchange; and mutualism (*M*), where metabolites are interchanged between the two models. Following this letter designation, a numerical value is used to represent the number of carbon sources added to the environment. Finally, the letters *a* or *b* are used to specify the absence and presence of competition, respectively. As an example, N1a would describe a simulation where a metabolite is taken up by only one cell in the presence of one carbon source. We used the same naming convention to classify our simulations (Fig. 4C). Firstly, with flux sampling, we notice substantially fewer instances of simulations where neither microbe achieved growth (204 compared to 114 of the 324 aerobic simulations and 246 compared to 114 of the 324 anaerobic simulations). At first glance, this is a counterintuitive result, as one might expect that it is more likely to achieve some growth without optimizing for it, rather than when attempting to maximize biomass. However, the result highlights the benefit of flux sampling. That is, due to the metabolic flexibility simulated with sampling, it is more likely that a microbe would secrete a metabolite beneficial for the other and thus enable the other cell to grow. In contrast, when each microbe is oriented towards maximizing its own growth, possibly at the expense of all other cellular goals (via FBA), that emergent interactive behavior is less likely. We also note differences between the aerobic and anaerobic conditions, with the aerobic sampling simulations producing more instances of cooperative mutualism (M1a: 90 with aerobic sampling vs 5 with anaerobic sampling), and the anaerobic simulations resulting in more non-competitive non-interaction (N1a: 91 with anaerobic sampling vs 0 with aerobic sampling).

## Discussion

Phenotypic metabolic heterogeneity, even in the monoculture of a genotypically uniform population, is known to have a substantial effect on observed community outcomes. However, the effects of this heterogeneity have been understudied, despite the rapid and substantial increases in modeling efforts at the genome-scale. In addition, microbes have been shown to exhibit sub-maximal growth, but this has not been widely studied using GEMs. While phenotypic heterogeneity and sub-maximal growth dynamics have been studied in individual GEMs of microbial activity, these two phenomena have not been analyzed for models of microbial interactions [31, 52–58]. In this work, we demonstrate how pairing disparate existing approaches of flux sampling and modeling of microbial communities advances the field of metabolic modeling. We systematically evaluate the impact of replacing FBA and its central assumption of maximal growth with a flux sampling approach.

We assess the effects of exploring the entire flux solution space with three distinct approaches of microbial community modeling: the compartmentalized approach, the lumped model or "enzyme soup" approach, and the costless secretion approach. With each approach, flux sampling replicates the major conclusions achieved with optimization of biomass using FBA. For example, we predict higher frequency of cooperation between microbes under anaerobic conditions. Furthermore, applying flux sampling expands our understanding of the systems-level heterogeneity that gives rise to observed community activity. For the compartmentalized approach, we show increased tendency toward stable consortia and provide an ability to identify distinct growth rate-dependent interaction regimes. For the lumped modeling approach, we predict large differences in the predicted flux for certain pathways and reactions and in the turnover of specific metabolites, when comparing FBA to flux sampling. When implementing flux sampling with the costless secretion approach, we predict a substantially wider range of metabolites secreted, enabling growth on substrates that had not been predicted when optimizing biomass using FBA.

As previously found, most observable metabolic heterogeneity across a population has two primary sources: variation in network structure and variation in network usage (divergence in form and functional utilization, respectively) [59]. Ensemble modeling of GEMs, accounting for variation in the structure of the metabolic network, has been shown to lead to increased accuracy and is of particular focus to the field with the emergence of novel tools; however, an equivalent effort has not been put towards understanding heterogeneous states achieved with a consistent network, despite the existence of flux sampling of GEMs as a tool for the past 20 years [60, 61]. To our knowledge, one paper has used sampling to study cell–cell metabolic interactions [62]. This gap has been identified by other researchers, and our work motivates future studies to more earnestly utilize and leverage the technique [63].

We recognize some limitations of our work. A particular weakness of genome-scale modeling is the difficulty in assigning constraints for the reaction fluxes. Without appropriate bounds on metabolic reaction rates, flux sampling may explore biologically unreasonable metabolic states. We have not directly compared the predicted growth rates to experimentally measured values. Rather, we consider the full range of flux distributions, given the stoichiometric and flux constraints. The emergence of novel experimental tools holds promises in addressing this limitation. For example, -omics technologies enable in vitro and in vivo measurements of growth rates, metabolite secretion, and impact of enzymatic knockouts. Such data can be used to provide biologically reasonable constraints on reaction fluxes. In addition, for the costless secretion approach, we used thresholding to keep the analyses computationally feasible. However, this potentially limits our results. Improvements in computational ability, both advances in computing speed and algorithm development, will enable us to investigate the full range of biological outcomes possible with flux sampling without imposing artificial thresholds. Additionally, we specifically compared traditional FBA with the flux sampling, but there are alternative approaches for analyzing microbial metabolic interactions. For example, parsimonious FBA (pFBA), flux variability analysis (FVA), hybridFBA (which uses nonparametric constraints), and unsteady-state FBA could all yield interesting insights into interactions between GEMs. Future work can apply such analyses to improve our understanding of microbial communities. Finally, by using flux sampling, we evaluated microbial fitness and interspecies relationships based on growth rate, while eliminating the necessity of maximizing biomass. Future work can explore alternative metrics to assess cellular behavior. This is especially important because genome-scale modeling is increasingly used for eukaryotic (principally, human) cells, where growth rate as a proxy for cell health is less supported [64–69]. For example, rather than focusing on growth, we could instead study flux through a specific reaction or pathway known to mediate the behavior of a particular cell type.

## Conclusion

In this work, we evaluate the effect of flux sampling on three standard approaches for modeling the interactions between microbes at the genome scale. Our results clearly distinguish between optimization-based and sampling-based characterizations of the metabolic interactions within a community. We demonstrate the utility of flux sampling in quantitatively studying metabolic interactions in microbial communities.

### Supplementary Information

**Additional file 1**. Supplementary figures S1 and S2.

## Data Availability

All required data and necessary code are available at the GitHub repository: https://github.com/FinleyLabUSC/FluxSampling_MetabolicInteractions.

## Abbreviations

FBA: Flux balance analysis
GEM: Genome-scale model
RHMC: Riemannian Hamiltonian Monte Carlo
