# Supplementary material for: Flux sampling in genome-scale metabolic modeling of microbial communities
Source: BMC Bioinformatics. 2024 Jan 29;25:45. doi: 10.1186/s12859-024-05655-3 (PMC10826046; doi:10.1186/s12859-024-05655-3)
Supplement: Supplementary file 1 — Additional file 1. Supplementary figures S1 and S2. [file 12859_2024_5655_MOESM1_ESM.docx]

**Flux sampling in genome-scale metabolic modeling of microbial communities**

Patrick E. Gelbach^a^, Handan Cetin^b^, and Stacey D. Finley^a,b,c^

^a^Alfred E. Mann Department of Biomedical Engineering, University of Southern California, Los Angeles, CA 90089, USA

^b^Department of Quantitative and Computational Biology, University of Southern California, Los Angeles, CA 90089, USA.

^c^Mork Family Department of Chemical Engineering and Materials Science, University of Southern California, Los Angeles, CA 90089, USA.

Corresponding Author: Stacey D. Finley, email - [sfinley@usc.edu](mailto:sfinley@usc.edu)

**Supplementary Information**


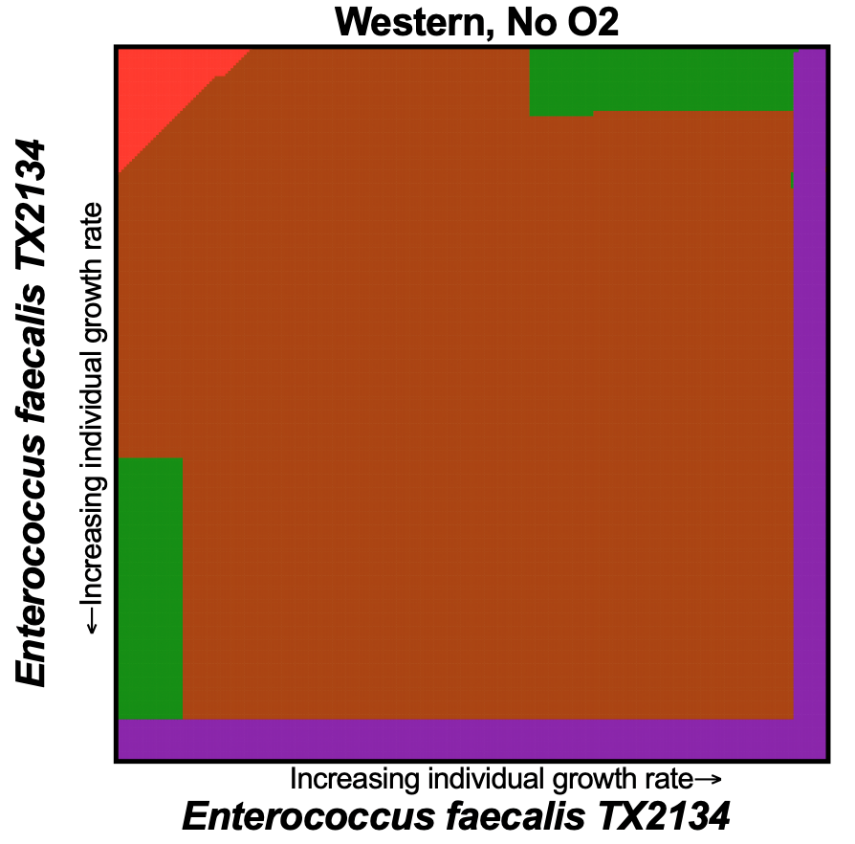


**Figure S1: Enterococcus faecalis TX2134 paired culture.** Expected interaction type when pairing two matching models of enterococcus faecalis, in an anoxic, Western Diet.

**
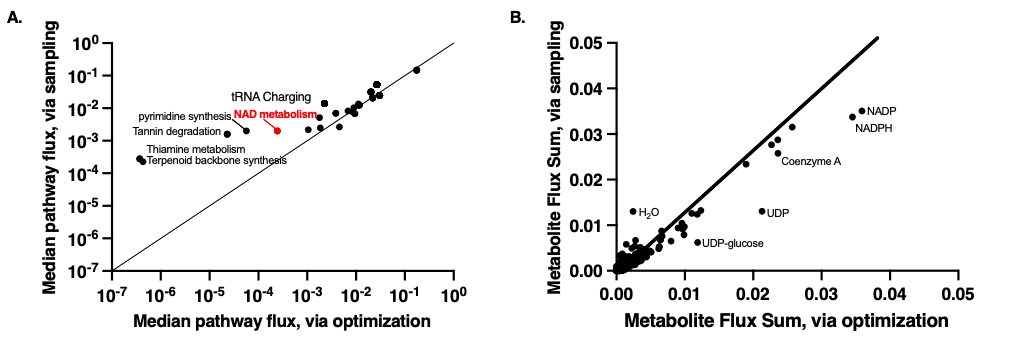
**

**Figure S2: Community-constrained sampling compared to FBA optimization. (A)** Median pathway flux values predicted by community-constrained flux sampling compared to optimization of biomass. Subsystems that have significantly different median fluxes are labeled. **(B)** Comparison of the flux-sum value for each metabolite for community-constrained flux sampling and optimization of biomass.
